# Sequencing Matters: Comparative Effectiveness of Density-Gradient Centrifugation and MACS for Enhancing Sperm Quality and DNA Integrity

**DOI:** 10.3390/jcm15155945

**Published:** 2026-07-30

**Authors:** Germar-Michael Pinggera, Elisabetta Piatti, Valentina Elisa Maria Pinggera, Marina Bellavia, Giovanni Maria Colpi

**Affiliations:** 1 Department of Urology, Medical University Innsbruck, 6020 Innsbruck, Austria; 2 Andrology and IVF Center, Next Fertility Procrea, 6900 Lugano, Switzerland; dottelisabettapiatti@gmail.com (E.P.); marina.bellavia@procrea.ch (M.B.); gmcolpi@yahoo.com (G.M.C.); 3 Faculty of Medicine, Danube Private University (DPU), 3500 Krems, Austria; valentinapinggera@gmail.com

**Keywords:** DNA fragmentation, spermatozoa, immunomagnetic separation, centrifugation, density gradient, sperm injections, intracytoplasmic, infertility, male, semen analysis, assisted reproductive technology

## Abstract

**Background**: Sperm DNA fragmentation (SDF) is increasingly recognized as a clinically relevant marker of male reproductive potential and has been associated with impaired embryo development, miscarriage, and suboptimal assisted reproductive technology outcomes, particularly in selected infertile populations. Because conventional sperm-preparation methods do not directly target molecular sperm damage, combined selection strategies such as density gradient centrifugation (DGC) and annexin V-based magnetic-activated cell sorting (MACS) have been proposed to enrich spermatozoa with improved DNA integrity before intracytoplasmic sperm injection (ICSI). Regardless of their more favorable functional characteristics, the optimal sequence for combining these methods remains uncertain. **Methods**: This study presents a hybrid analysis combining a structured review of 25 published studies with original prospective observational laboratory data. The review examined the effects of DGC, MACS, and sequential combined protocols on SDF, conventional semen parameters, and reported assisted reproduction outcomes, with particular attention to the unresolved question of sequencing order. In parallel, a blinded observational cohort of 67 infertile men with baseline SDF of at least 30% underwent sequential DGC followed by MACS, with SDF measured before and after processing using the sperm chromatin dispersion test (Halosperm ^®^ assay). **Results**: Across the reviewed literature, combined DGC/MACS approaches generally reduced SDF more than single-step preparation, although the magnitude of benefit varied across assays, patient populations, and laboratory protocols. Some studies also reported improvements in embryo quality and pregnancy-related outcomes, but these data were derived mainly from retrospective or small comparative cohorts, and no large randomized head-to-head trials were identified comparing DGC→MACS with MACS→DGC for fertilization, miscarriage, or live-birth outcomes. In the observational cohort, DGC→MACS was associated with a significant mean absolute SDF reduction of 19.42% +/− 9.4% (*p* < 0.0001). Most patients (85.1%) achieved post-treatment SDF values below the 30% threshold. **Conclusions**: Combined DGC/MACS sperm preparation, supported by the data in our study sequence, appears promising for reducing SDF in selected infertile men, particularly those with elevated baseline SDF. However, the optimal sequencing strategy and its impact on major reproductive endpoints remain uncertain and require better standardized comparative clinical studies.

## 1. Introduction

Sperm DNA fragmentation (SDF) has emerged as an important functional marker in the evaluation of male infertility because conventional semen analysis provides limited information about sperm genomic integrity and reproductive competence [1,2]. Elevated sperm DNA fragmentation has consistently been associated with an increased risk of miscarriage [3,4,5,6]. In a landmark meta-analysis including 16 cohort studies and 2969 couples, Robinson et al. demonstrated that high sperm DNA damage was associated with a more than twofold increase in miscarriage risk (RR 2.16, 95% CI 1.54–3.03) [7], confirmed in one other recent umbrella SRMA with similar RR [8], whereas its effects on fertilization and embryo development appear more variable across studies. The magnitude of these effects varies according to the assay used, study population, and fertilization method [1,9,10]. Within ART, the clinical relevance of SDF appears particularly important in couples with recurrent reproductive failure, unexplained infertility, poor semen quality, and selected male-factor phenotypes [9]. Elevated SDF has been linked more consistently to adverse outcomes in intrauterine insemination and conventional in vitro fertilization than in intracytoplasmic sperm injection (ICSI), where some negative effects may be attenuated but not necessarily eliminated [6,10,11]. A large meta-analysis of IVF/ICSI cycles by Ribas-Maynou and colleagues concluded that sperm DNA damage negatively influences implantation and clinical pregnancy when IVF and ICSI are pooled, with stronger effects in conventional IVF than in ICSI [6]. More recent umbrella syntheses and guidelines similarly acknowledge that high SDF correlates with poorer reproductive outcomes but also emphasize assay heterogeneity and moderate quality of evidence [3,4,5,12,13]. This distinction is clinically relevant because ICSI bypasses natural barriers to sperm selection while still exposing the embryo to the biological consequences of sperm nuclear damage [11].

Several sperm-preparation techniques have therefore been developed or refined to enrich spermatozoa with better structural and functional characteristics before ART [9,11]. Density gradient centrifugation (DGC) remains one of the most widely used laboratory methods and is effective in separating motile, morphologically superior sperm from debris, leukocytes, and dead cells [2]. However, DGC does not directly target molecular markers of apoptosis or DNA injury, and in some settings its ability to reduce SDF is incomplete or variable, particularly in samples with poor baseline semen quality or high pre-treatment DNA damage [2,14,15,16].

Magnetic-activated cell sorting (MACS) using annexin V was introduced to address this limitation by depleting spermatozoa that externalize phosphatidylserine, a membrane feature associated with apoptosis [9,17,18]. MACS can enrich the final sperm fraction with non-apoptotic cells that tend to have lower levels of DNA fragmentation, and several studies have shown additional benefit when MACS is combined with DGC or swim-up rather than used alone [9,14,19,20]. More recently, systematic review evidence has supported the capacity of MACS-based selection to reduce sperm DNA damage and to improve selected reproductive outcomes in medically assisted reproduction, especially in populations with elevated baseline SDF [11].

Despite the growing literature, an important procedural question remains unresolved: whether MACS should be performed before DGC or after DGC. Comparative studies have not yielded uniform conclusions. Tavalaee et al. favored MACS before DGC on the basis of laboratory markers related to apoptosis and chromatin maturity, whereas Bucar et al. reported the greatest SDF reduction with a MACS→DGC→swim-up sequence in selected samples [14,15]. By contrast, several clinically oriented studies reporting favorable embryo or pregnancy outcomes used protocols in which DGC preceded MACS, including the retrospective study by Pacheco et al. in couples with elevated SDF [9]. Because these studies differ in design, comparator groups, semen characteristics, and outcome measures, the literature does not currently establish one universally superior sequencing strategy [9,11,14,15].

### 1.1. Clinical Translation

The uncertainty is not only technical but also clinical. Combined sperm-selection protocols appear promising for reducing SDF, but the translation of improved laboratory parameters into fertilization, embryo development, miscarriage reduction, and live birth remains supported mainly by retrospective or small comparative studies rather than large randomized head-to-head trials [9,11,21,22,23]. This creates a risk of overinterpreting laboratory gains as proof of clinical superiority, particularly when one sequence is favored over another without direct comparative outcome data [11].

### 1.2. Patient Selection

Another unresolved issue concerns patient selection. Published studies indicate potential advantages of combined sperm-selection strategies in men with high baseline SDF, asthenozoospermia, asthenoteratozoospermia, selected immotile but viable sperm populations, and certain cryopreservation settings [9,14,20,21,22,24,25,26]. However, these observations derive from heterogeneous cohorts, largely on laboratory and subgroup data, and further studies are needed to confirm their relevance to clinical outcomes. Identifying which patients are most likely to benefit from an additional selection step therefore remains a clinically important goal [9,11].

Against this background, the present manuscript was designed as a hybrid study with two complementary aims. First, it provides a structured review of published studies evaluating DGC, MACS, and combined sequencing strategies with regard to SDF reduction, semen quality, and reported reproductive outcomes. Second, it presents original prospective blinded observational laboratory data from infertile men with baseline SDF ≥ 30% undergoing sequential DGC followed by MACS. By integrating the published literature with original cohort data, the study seeks to clarify what can be concluded with reasonable confidence about combined sperm selection, what remains uncertain regarding sequencing, and which clinical scenarios may justify selective use of a DGC/MACS approach.

## 2. Overview of Sperm-Selection Techniques to Reduce DNA Fragmentation

Sperm DNA fragmentation is an important marker in the evaluation of male infertility, as elevated SDF has been associated with reduced fertilization rates, impaired embryo development, increased miscarriage risk, and lower live-birth rates in ART cycles. Accordingly, sperm-selection protocols aimed at reducing DNA fragmentation may improve laboratory and, in selected settings, clinical outcomes. Therefore, optimization of sperm-selection protocols with respect to DNA fragmentation may help better treat infertile couples, particularly when standard semen analysis remains inconclusive or for problematic ART attempts. A range of techniques is currently employed for this purpose and can be summarized as follows:

### 2.1. Density Gradient Centrifugation Protocol and Efficacy

**Figure 1:**
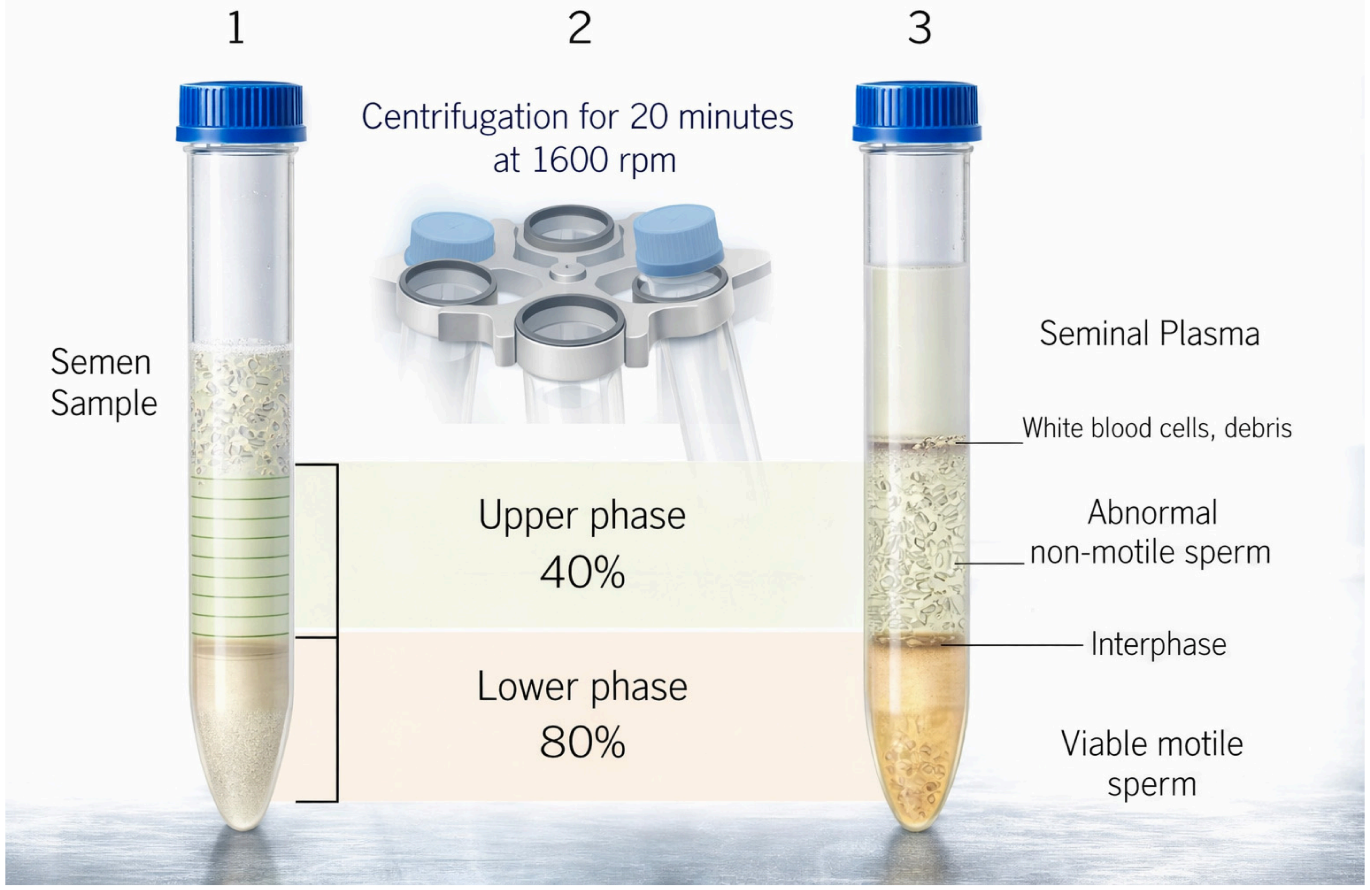
Sperm separation technique by DGC after centrifugation at 1600 rpm (400× *g*). [Picture refined by AI-generated app using Google AI Studio Gemini 3, Nano Banana Pro: 28 December 2025].

Density gradient centrifugation (DGC) is a standard sperm-preparation technique that exploits differences in cellular density to isolate a highly motile and morphologically normal sperm fraction. Typically, semen is layered onto a discontinuous gradient, most commonly comprising 40% and 80% density layers, and centrifuged at 300–500× *g* for 15–20 min. This approach efficiently removes non-viable spermatozoa, cellular debris, leukocytes, and other contaminants, thereby improving sample quality for downstream diagnostic and assisted reproductive procedures [27,28,29]. Although DGC generally reduces sperm DNA fragmentation relative to unprocessed semen, some samples paradoxically exhibit increased SDF after processing, particularly in men with high baseline SDF or poor semen quality. Thus, DGC reliably improves motility and morphology; however, its ability to reduce SDF is lower than that of the swim-up method in samples with <30% SDF [2]. When combined with MACS, the reduction in DNA fragmentation becomes significantly greater than when the technique is used alone [9,14,15,24]. The sequential steps involved in sperm separation by density gradient centrifugation (DGC) are illustrated in Figure 1.

### 2.2. Swim-Up Technique Protocol and Efficacy

**Figure 2:**
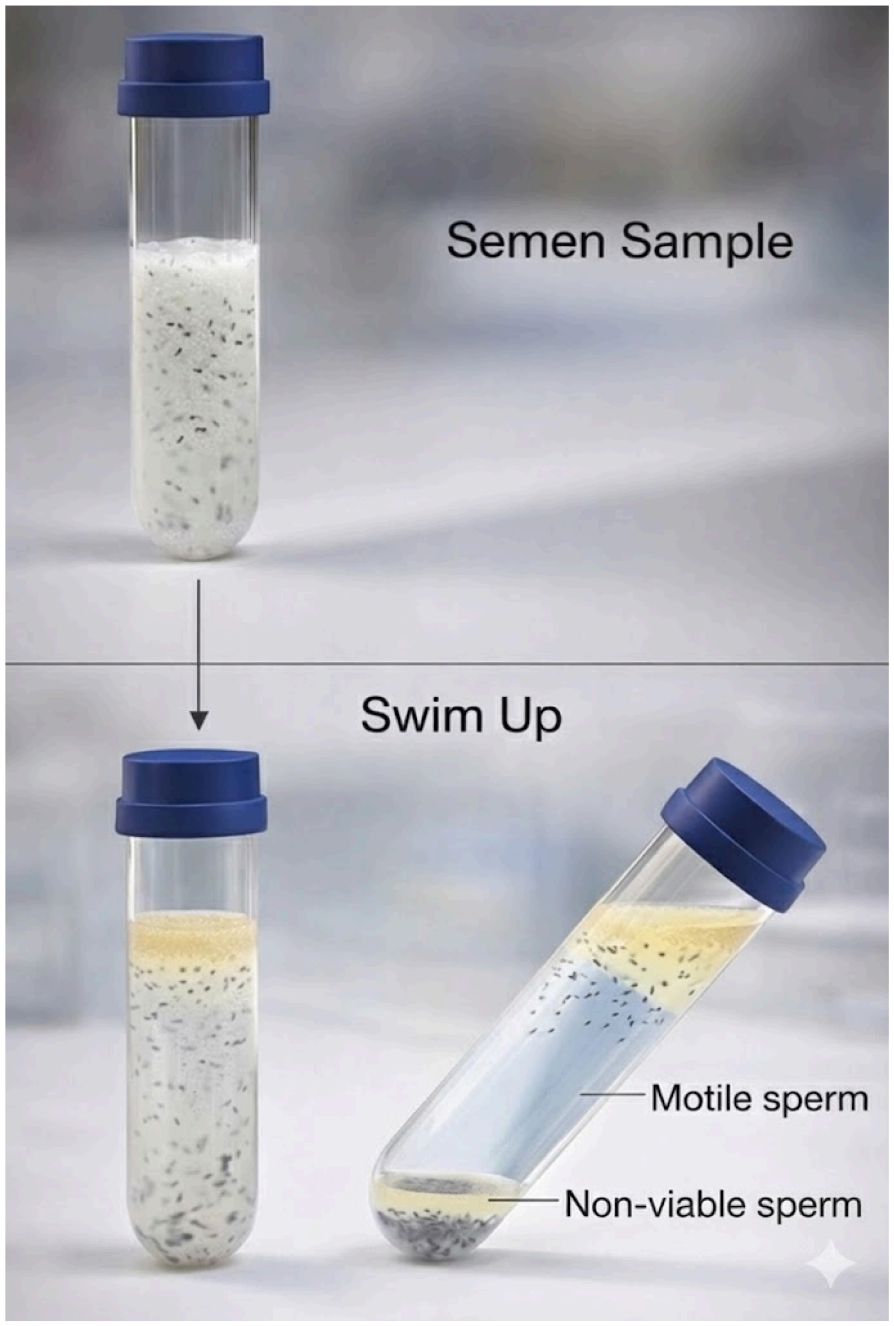
Demonstration of the 45° slope swim-up technique for semen clearance. [Picture refined by AI-generated app using Google AI Studio Gemini 3, Nano Banana Pro 28 December 2025].

The swim-up procedure typically involves overlaying liquefied semen with culture medium and incubating it at 37 °C for 30–60 min. Motile spermatozoa swim into the medium, which is then collected for use [2,30,31]. The 45° inclined swim-up technique used for semen processing and motile sperm recovery is illustrated in Figure 2. Some studies have reported that swim-up enriches motile sperm and may yield favorable DNA-fragmentation outcomes with a DFI below 30% under selected conditions, although results vary by sample quality and assay. Compared with DGC, swim-up may produce a higher immediate motile fraction, but post-processing survival can be lower [2,32]. However, the percentage that survives after 24 h is lower in comparison with DGC [2]. Optimized protocols, such as the one-step swim-up/ICSI approach, which reduces, and in some cases even completely eliminates the need for centrifugation, can select a sperm population with near-zero SDF for ICSI. Therefore, such a procedure can outperform the conventional swim-up technique and, in some instances, even achieve better results than MACS [31].

### 2.3. Magnetic-Activated Cell Sorting (MACS) Protocol and Efficacy

**Figure 3:**
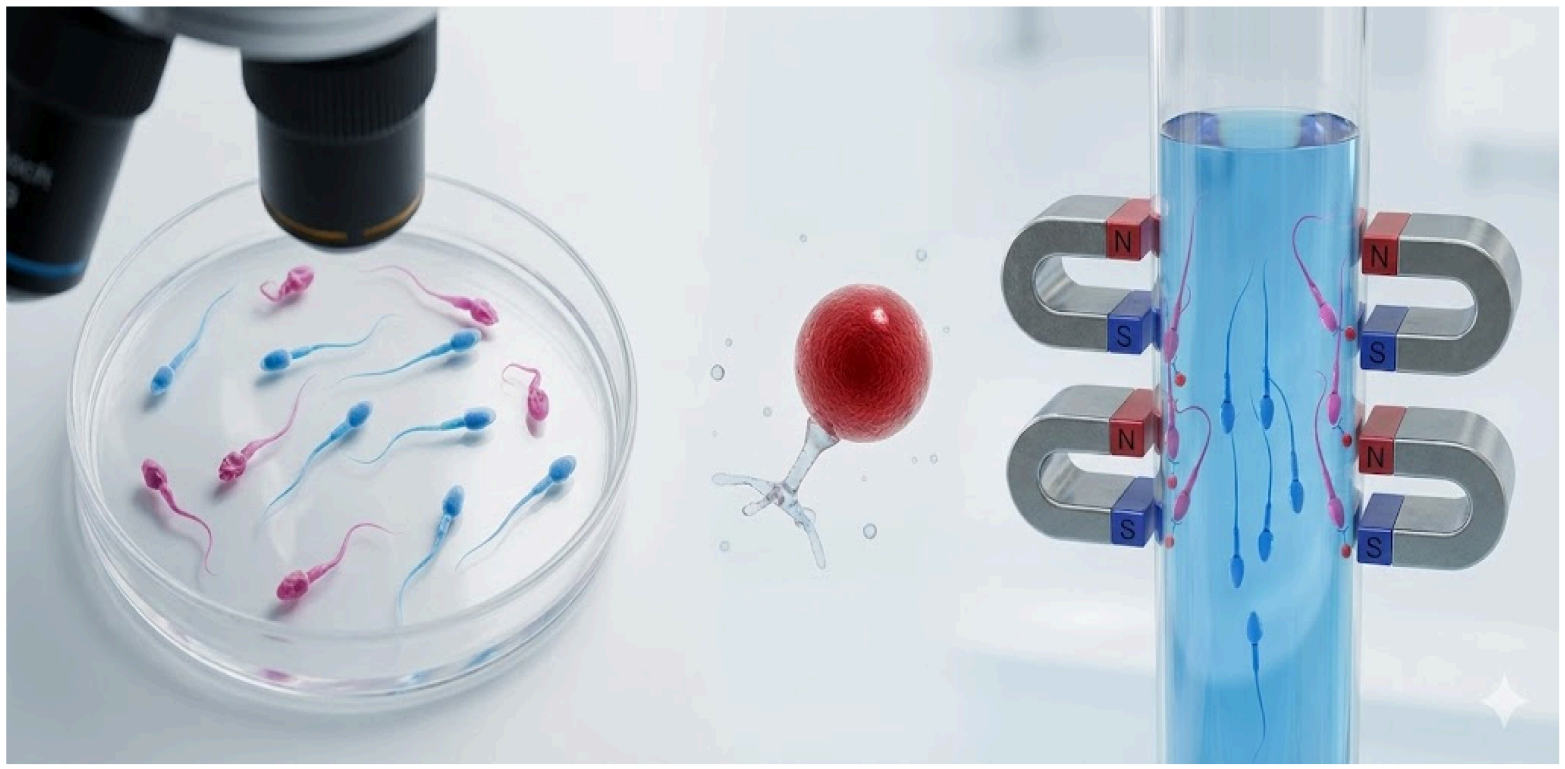
Principle for magnetic-activated cell sorting (MACS) technique. In this schematic, the colors distinguish sperm populations according to their membrane status during MACS: Blue spermatozoa represent viable, non-apoptotic sperm cells that do not expose phosphatidylserine and therefore pass through the magnetic column. Pink spermatozoa represent apoptotic or membrane-compromised sperm cells with externalized phosphatidylserine; after binding annexin V–coated magnetic microbeads, they are retained within the magnetic field. [Picture refined by AI-generated app using Google AI Studio Gemini 3, Nano Banana Pro 28 December 2025].

MACS uses annexin V-conjugated magnetic beads for the depletion of apoptotic sperm by binding phosphatidylserine-externalizing cells, thereby enriching the final preparation with non-apoptotic, DNA-intact sperm [9,11]. The principle of magnetic-activated cell sorting (MACS), whereby annexin V–coated magnetic microbeads retain phosphatidylserine-exposing apoptotic spermatozoa while the unbound viable sperm fraction passes through the magnetic column, is illustrated in Figure 3. MACS is most effective in patients with high SDF, and when combined with DGC or swim-up, it can further reduce DNA fragmentation beyond the levels achieved by conventional methods [9,11]. In selected patients with high SDF, MACS-containing protocols have been reported to further reduce DNA fragmentation beyond conventional methods, especially when combined with DGC or swim-up [9,11]. Bucar et al. reported the greatest reduction in DNA fragmentation with the MACS-DGC-SU sequence, in which MACS is applied to raw semen followed by DGC and then swim-up [14]. Although this approach provides the most substantial decrease in SDF, it may compromise sperm vitality and motility in samples with poor baseline parameters [14]. Nevertheless, the optimized protocol remains under expert review.

## 3. Materials and Methods

### 3.1. Structured Review of the Literature

The literature search was conducted to identify studies evaluating sperm DNA fragmentation before and after sperm preparation, with specific attention to the sequence and timing of DGC and annexin V-based MACS procedures. Randomized controlled trials, controlled trials, cohort studies, before–after studies, and cross-sectional human studies published in English were screened and included, as summarized in Table 1.

The review aimed to compare DGC alone, MACS alone, sequential combinations of DGC and MACS, and protocols incorporating swim-up. Extracted variables included total sperm count or concentration, total motile sperm count, progressive motility, morphology, vitality, semen volume, baseline SDF/DFI values, post-treatment SDF/DFI values, and fertility outcomes where reported. Data on statistical testing, including paired t tests, Wilcoxon signed-rank tests, *p*-values, confidence intervals, effect sizes, and power analyses when available, were also collected.

Twenty-five primary studies examining combined DGC and MACS for sperm selection in assisted reproduction were identified. These studies spanned from 2006 to 2023 and were heterogeneous with respect to design, patient population, assay platform, and clinical indication.

### 3.2. Observational Study Design

Original data were generated in a prospective blind observational study including 67 male patients undergoing infertility evaluation, with a mean age of 43.16 +/− 7.4 years (range 30–66 years). The primary inclusion criterion was pathological sperm DNA fragmentation, defined in this cohort as a baseline SDF value of at least 30% [19,22]. All semen samples underwent a standardized two-step preparation protocol. First, samples were processed by discontinuous DGC using 80% and 40% gradients, followed by centrifugation at 400× *g* for 20 min and a wash step at 500× *g* for 5 min, in line with conventional DGC methods used in ART laboratories [2,19]. Second, the post-gradient sample underwent annexin V-based MACS according to the Miltenyi Biotech kit workflow, including incubation with annexin V-conjugated microbeads and subsequent magnetic-column separation [18,43]. After elution, the sperm fraction was washed with HTF medium, centrifuged at 500× *g* for 5 min, and resuspended in fresh medium before analysis, following standard MACS protocols [19]. For each patient, SDF was measured before and after treatment using the sperm chromatin dispersion test (Halosperm assay), a validated method widely employed in both MACS and DGC evaluation studies [2,19]. Repeat semen analysis was performed according to the WHO 6th edition at both time points, and all assessments were conducted blindly [29].

The present study used only data generated during routine fertility evaluations performed in the course of standard clinical care following careful patient information. No additional study-related interventions, examinations, or procedures were undertaken. All data were fully anonymized before analysis, and formal IRB approval was therefore not required. Where indicated, selected illustrations were developed from original photographs or manually drawn artwork and were subsequently modified and optimized with the assistance of artificial intelligence-based tools. All outputs were produced under author supervision and were critically reviewed, corrected, and approved by the authors before inclusion in the manuscript.

**Figure 4:**
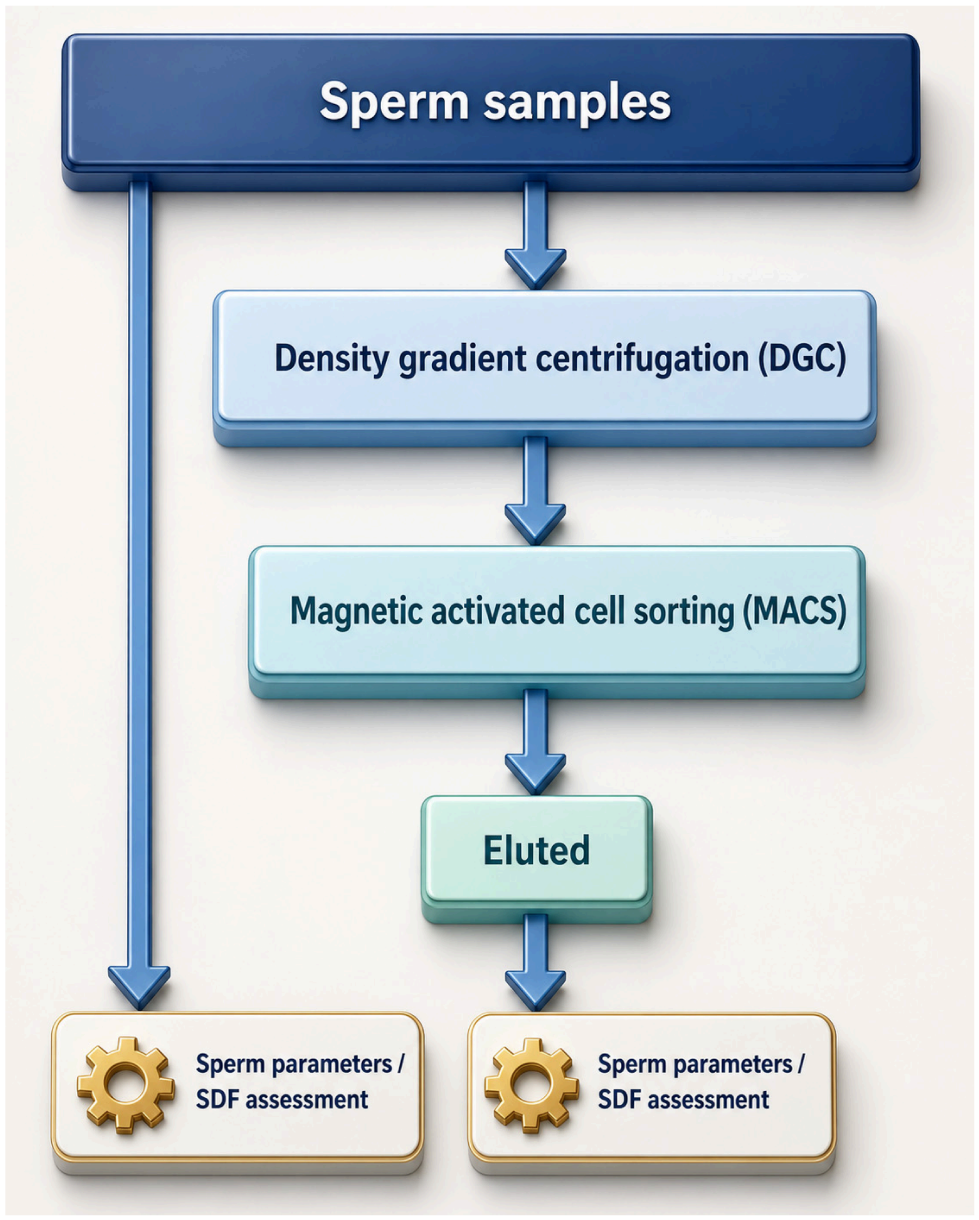
Schematic workflow of sperm sample processing. Schematic workflow of sperm sample processing by density gradient centrifugation (DGC) followed by magnetic-activated cell sorting (MACS), with sperm parameters and sperm DNA fragmentation (SDF) assessed before and after processing. The diagram shows the sequential handling of sperm samples through DGC, MACS, and elution, with endpoint assessment of sperm quality and DNA integrity.

The semen preparation workflow is shown in Figure 4.

### 3.3. Outcomes and Statistical Analysis

The primary endpoint of the observational study was the change in SDF after sequential DGC followed by MACS, intended to compare primary laboratory endpoints by prior MACS and DGC-MACS investigations [9,14]. Pre- versus post-treatment comparisons were assessed using the Wilcoxon signed-rank test, a non-parametric method appropriate for skewed paired data in small-to-moderate samples, commonly used in sperm-preparation studies. The analysis also reported *p*-values for primary comparisons, and confidence intervals or effect sizes when available [14].

## 4. Results of the Structured Review

### 4.1. Characteristics of Included Studies

The 26 included studies were highly heterogeneous in design, population, and outcome assessment. Sample sizes ranged from 15 patients or semen samples to 724 treatment cycles [9,10]. The studies included men with varicocele, oligoasthenoteratozoospermia, asthenozoospermia, teratozoospermia, unexplained infertility, cryopreserved samples, chromosomal abnormalities, and elevated baseline SDF [20,25,37].

The studies also used different assays to assess sperm DNA damage, including TUNEL, sperm chromatin structure assay (SCSA), and sperm chromatin dispersion methods such as Halosperm [6]. Prior reviews have shown that these assays are not directly interchangeable because effect sizes and threshold performance differ substantially across methods. Assay-related variability in cut-offs, reporting formats, and interpretation further limits direct comparison of absolute DFI values across studies. This methodological heterogeneity is known to influence absolute DFI values and should be considered when comparing absolute reductions across studies [12]. In a large global survey across experts, a notable variability in assay use, reporting formats, and different cut-offs across centers was found, all of which complicate inter-study comparison [3].

### 4.2. DNA Damage Assays and Protocol Sequences

Different DNA fragmentation assessment methods were used across the included studies, including TUNEL assay [9,14,16,20,35,36], sperm chromatin structure assay (SCSA) [33], the sperm chromatin dispersion technique (SCD) [21,39], and the Halosperm method [19]. Because these assays measure DNA damage differently and use non-equivalent thresholds, absolute values should not be pooled or compared directly across studies.

The reviewed literature also used several sperm-preparation sequences, most commonly DGC followed by MACS and, less frequently, MACS followed by DGC or combinations incorporating swim-up [44]. These protocol differences reflect laboratory practice, semen characteristics, and study-specific aims, and they limit direct cross-study comparison. An overview of the evaluated sperm-preparation protocols, including DGC→MACS, MACS→DGC, swim-up-based combinations, and comparative protocols, is provided in Table 2.

DGC-first protocols were employed in studies by Degheidy, Sanchez-Martin, Toishibekov, Zhang, Chi, Pacheco, Lee, Delbes, Esbert, Said, Bibi, and Merino-Ruiz [9,10,18,19,20,24,25,33,37,39,40]. MACS-first approaches were used in studies by Tavalaee [15], Salehi Novin, Fang, and Ziarati, which investigated MACS→DGC or MACS→DGC→ICSI workflows [15,21,23,34]. The presence of different sequencing models across the literature underscores that no single preparation algorithm has been uniformly adopted. Instead, protocols vary according to laboratory practice, semen quality, and study-specific aims [16,44].

### 4.3. Effects on Sperm DNA Fragmentation

Across studies, combined sperm-selection approaches generally reduced SDF more than single-step procedures. Reported reductions varied by study, assay, and patient population, but multi-step or MACS-containing protocols consistently showed meaningful decreases in DNA fragmentation-related endpoints. Reported absolute reductions ranged from approximately 2.8 to 21.9 percentage points, while relative reductions in DNA-fragmented sperm ranged from roughly 39% to over 80% [14,25,33]. Chi and colleagues reported a stepwise decline in DFI from DGC alone to MACS alone and then to combined DGC+MACS, suggesting an additive effect of sequential processing; DGC alone reduced DFI from 11.5% to 8.1%, MACS alone to 7.4%, and combined DGC→MACS further to 4.1%, suggesting an additive effect of combined processing [19]. Zhang et al. similarly found lower post-treatment DFI with DGC→MACS than with DGC alone in men with immotile but viable spermatozoa [24]. In the Bibi study, baseline SDF differed slightly among the four preparation groups, but post-preparation sperm DNA fragmentation (SDF) was significantly lower in the DGC→MACS group (12.3%) than in DGC, SU, and DGC-SU (14.7%, 14.5%, and 14.2%, respectively) [39].

Sequence-specific findings were not uniform. In direct comparisons, some studies favored MACS→DGC over DGC→MACS for selected apoptosis-related endpoints, while others found the opposite for specific protocol combinations. Tavalaee et al. found that MACS→DGC provided greater reductions in active caspase-positive and TUNEL-positive sperm compared with DGC→MACS in a small direct comparison [15]. Bucar et al. reported the greatest relative reduction with a MACS→DGC→swim-up workflow in their cohort (−83.3%), whereas DGC→swim-up→MACS performed least well [14]. Berteli et al. also observed lower median post-processing DFI with MACS→DGC than with DGC→MACS in donor samples [38]. The sperm DFI decreased from a median of 24% at baseline to 10% after DGC, 6% after DGC-MACS, and 4% after MACS-DGC [38]. Taken together, these studies suggest that combined treatment strategies are effective in reducing SDF; however, the available evidence does not support the superiority of any single treatment sequence across all clinical scenarios and laboratory settings. The effects of different sperm-preparation sequences, including DGC, MACS, swim-up (SU), and combined protocols, on the DNA fragmentation index are summarized in Table 3.

### 4.4. Effects on Conventional Sperm Parameters

Several studies reported favorable changes in sperm motility, viability, and membrane integrity after combined preparation. Toishibekov et al. found an increase in motility from 32.7% to 47.2% in the annexin-negative fraction [33]. Zhang et al. found that DGC+MACS enriched viable sperm with intact membranes and lower DNA fragmentation in immotile populations [24]. However, Cakar et al. showed that adding MACS after DGC or swim-up significantly reduced total sperm concentration and rapid progressive motility, especially in oligozoospermic men, leading the authors to caution against routine use of MACS in all cases [16]. These findings underscore that the clinical significance of SDF reduction should be evaluated in the context of sperm recovery, especially in men with low sperm counts, where optimization of DNA integrity may need to be balanced against sperm yield. The study-level effects of combined sperm-preparation protocols on sperm parameters across different clinical conditions are summarized in Table 4.

### 4.5. Chromatin Quality and Chromosomal Abnormalities

Some studies reported improvements in markers related to chromatin packaging and chromosomal abnormality after combined preparation. Chi et al. found lower protamine deficiency after DGC plus MACS than after DGC or MACS alone [19], while Delbes et al. demonstrated that annexin V-based selection enriched sperm quality in asthenoteratozoospermic and teratozoospermic samples, as assessed by complementary assays of chromatin status [20]. Additional studies suggested that both DGC and MACS may reduce the proportion of sperm with chromosomal abnormalities. Brahem et al. reported that DGC reduced the proportion of sperm with aneuploidy in subfertile men [45]. Rouen et al. showed that discontinuous gradient centrifugation decreased the proportion of chromosomally unbalanced spermatozoa in carriers of structural chromosomal rearrangements [46], while Esbert et al. observed that spermatozoa with numerical chromosomal abnormalities were more likely to be retained in annexin V–MACS columns than in the eluted fraction [37]. El Fekih et al. further reported that MACS–annexin V sorting of semen with high TUNEL values decreased the concentration of sperm with abnormal chromosomal content [41]. These findings are relevant and promising but derive from specialized cohorts and surrogate laboratory endpoints rather than uniform clinical outcome studies.

### 4.6. Clinical ART Outcomes

Clinical outcome data were more limited, and clinical outcomes were variable across studies or more heterogeneous than laboratory SDF data suggest. Fertilization rates were often similar between combined-preparation and control groups, whereas some studies reported better embryo quality, implantation, pregnancy, miscarriage, or live-birth outcomes after MACS-containing protocols. For example, Salehi Novin et al. reported no meaningful fertilization difference but higher top-quality embryos and blastocyst rate with DGC→MACS [21]. In a prospective study of 196 ICSI cycles, MACS-selected spermatozoa before DGC were associated with a higher chemical pregnancy rate than DGC alone, 61.47% versus 45.95% [42]. The same study also reported a higher clinical pregnancy rate in the MACS group, 48.36% versus 36.49%, although this difference did not reach conventional statistical significance (*p* = 0.052). The cleavage rate was significantly higher after MACS selection, 97.2% versus 88.2% in the DGC group (*p* < 0.01), but not the implantation rate, as well as the fertilization rate, with 69.52% in the MACS group and 69.9% in the DGC group [42]. Notably, this study did not report the difference in SDF for both techniques used.

Pacheco et al., in the largest retrospective cohort of 724 cycles involving men with elevated SDF, also support the statement that DGC→MACS was associated with a better outcome, although fertilization rate was not statistically significant, with 75.1% vs. 73.3% (*p* = 0.13) [9]. However, he also reported higher pregnancy rates (60.7% vs. 51.5%, *p* = 0.0014), lower miscarriage rates (14.7% vs. 20.6%; *p* = 0.03), and higher live-birth rates (47.4% vs. 31.2%; *p* = 0.001) in the group treated with DGC followed by MACS compared with DGC alone, while fertilization rates did not differ significantly [9]. Mei et al. observed that MACS in high-DFI patients improved live-birth rates per initiated cycle and reduced the number of embryo transfers required per live birth, and thus their data support the claim of better live-birth-related outcomes and fewer transfers per live birth [22], while Salehi Novin and Ziarati reported better embryo-quality outcomes and implantation in MACS→DGC groups versus DGC alone in high-DFI male-factor cohorts [21,23]. Notably, the Notrica et al. study appears inconsistent with the broader data, because it reports no significant improvement in fertilization or pregnancy in ICSI, which is important negative evidence [35]. Bibi et al. (2023) found in a single prospective study with four preparation arms that DGC-MACS improved sperm quality, cleavage rate, and clinical pregnancy (DGC→MACS 52.5%, DGC 44.4%, SU 35.8%, DGC→SU 36%) in 395 teratozoospermic couples [39]. In this study, DGC→MACS improved cleavage rate and was associated with the highest pregnancy rate, while fertilization and implantation rates were broadly comparable across preparation methods.

These findings are clinically important, but they should be interpreted cautiously. Most available evidence for pregnancy and live-birth outcomes comes from retrospective cohorts or relatively small comparative studies, and no large randomized head-to-head trials comparing DGC→MACS with MACS→DGC for fertilization, pregnancy, or live-birth endpoints were identified in the reviewed literature [11]. Embryological and clinical ART outcomes following DGC, DGC–MACS, and related sperm-selection protocols—including fertilization, embryo quality, pregnancy, miscarriage, and live-birth rates—are summarized in Table 5.

### 4.7. Clinical Subgroups Most Likely to Benefit

The reviewed studies suggest that combined sperm-selection methods may be most relevant in selected clinical populations rather than as a universal intervention. The literature does support that these methods tend to be most useful in men with elevated SDF, certain motility defects, or specific sperm functional abnormalities, rather than in all infertile men.

The clearest subgroup is men with elevated baseline SDF, particularly those with values at or above 30%, because several studies specifically enrolled such patients and reported meaningful reductions in SDF and, in some cohorts, improved downstream reproductive outcomes [9,19,21,22].

Evidence also suggests possible benefit in patients with asthenozoospermia or asthenoteratozoospermia, where MACS-containing or combined protocols appear to yield larger relative reductions in DFI than in isolated teratozoospermia [14,20]. Bucar et al. reported that the highest SDF reduction occurred when MACS was applied before DGC and swim-up, and that this effect was negatively correlated with progressive motility, vitality, and membrane integrity, while teratozoospermic patients tended to have lower reduction rates than asthenozoospermic and asthenoteratozoospermic patients [14]. Additional potentially relevant subgroups include patients with immotile but viable sperm and selected cryopreservation settings, although evidence in these contexts remains limited and study-specific [24]. Potential baseline determinants of response to sperm-selection procedures—including initial semen quality, diagnostic category, baseline DFI, PLCζ expression, apoptotic markers, and sperm morphology—are summarized in Table 6.

In summary, combined sperm-selection methods appear most useful in selected cases, particularly men with elevated SDF/DFI, impaired motility or vitality, and specific apoptotic or chromatin abnormalities. In these groups, MACS-containing protocols often reduce DNA fragmentation and may improve embryo development, while evidence for pregnancy and live-birth benefit remains study-specific and less consistent. However, evidence in these niche contexts remains limited and highly study-specific.

## 5. Results of the Observational Study

### 5.1. Cohort and Primary Outcome

The observational cohort included 67 infertile men with a mean baseline SDF of 41.61 +/− 11.2% and a median of 38%, consistent with high-DFI populations in prior MACS studies [22]. After sequential DGC followed by MACS, the mean absolute reduction in SDF was 19.42 ± 9.4%, with a median reduction of 19% and a range of 4% to 43%.

The pre- to post-treatment decrease in SDF was highly significant on Wilcoxon signed-rank testing (*p* < 0.0001), indicating a substantial laboratory effect of the combined protocol in this selected cohort. This corresponds to an approximate 45% to 50% relative reduction from baseline. This magnitude of reduction is comparable to or greater than those reported in other DGC/MACS series employing similar inclusion criteria [14,19]. The findings are summarized in Table 7.

### 5.2. Responder Categories

Most patients responded favorably to treatment. Fifty-seven of 67 patients (85.07%) achieved post-treatment SDF values below 30% and were classified as adequate responders. In this subgroup, the mean SDF reduction was 20.65 ± 9.45%. Ten patients (14.92%) remained at or above 30% SDF despite a measurable absolute reduction and were categorized as inadequate responders, with a mean reduction of 12.4 ± 5.4%. Six patients (8.95%) showed near-complete elimination of fragmented sperm and were described as exceptional responders. Overall, these patterns suggest that patients with baseline SDF ≥ 30% may derive greater benefit from MACS-containing protocols. These findings are broadly consistent with prior data showing greater relative SDF reduction in high-DFI ejaculates [6].

### 5.3. Baseline Predictors

Linear regression showed no significant correlation between the magnitude of SDF reduction and native total sperm count or total motile sperm count (*p* > 0.1). This suggests that elevated SDF may occur independently of conventional semen parameters. Infertile men with otherwise normal semen parameters may still exhibit pathologically high SDF, supporting other studies [48,49]. Therefore, treatment response cannot be inferred solely from baseline sperm count metrics.

## 6. Discussion

This manuscript combined a structured review of the literature with a prospective blinded observational cohort to evaluate whether sequential sperm selection with DGC and MACS can improve sperm DNA integrity in infertile men. The principal finding is that combined sperm-selection approaches consistently reduce SDF across heterogeneous studies, and that in the present cohort sequential DGC followed by MACS was associated with a marked and statistically significant mean absolute reduction in SDF of 19.42%. These findings support the laboratory value of combined sperm preparation in men with elevated baseline SDF, but they do not establish that one sequence is definitively superior for clinical outcomes.

The most consistent signal across the reviewed studies concerns laboratory improvement in DNA integrity. Multiple reports showed that combining DGC and MACS lowers SDF more effectively than DGC alone and, in some settings, more effectively than MACS alone [9,14,15,19,24,25]. This pattern was particularly evident in studies such as Chi et al., where the combined approach outperformed every single technique, and in studies focused on poor-quality or high-DFI samples [19,25]. These data support the concept that DGC and MACS may act in a complementary rather than redundant manner, with DGC enriching for motile and morphologically superior spermatozoa and MACS further depleting annexin V-positive cells more likely to carry DNA damage [9,18,19]. On the other hand, some smaller or methodologically limited studies show no clear clinical benefit despite SDF changes. Notrica et al., 2013, reported comparable fertilization and pregnancy despite improved laboratory parameters [35], and Ziarati et al., 2019, reported similarly improved embryo quality and some implantation benefit but nevertheless did not report that advanced sperm sorting resulted in dramatic differences in live-birth rates across all subgroups [23].

The present observational data are concordant with the broader laboratory literature. In men selected for baseline SDF ≥ 30%, DGC→MACS produced a highly significant reduction in DNA fragmentation, and most patients crossed below the pre-specified pathological threshold after treatment. This is clinically relevant because elevated baseline SDF is one of the main situations in which advanced sperm-selection strategies are considered in current expert guidance [9]. The response pattern also suggests that combined preparation may be particularly useful in men with substantial pre-treatment DNA damage, even though the present cohort did not include a non-combined comparator arm. Accordingly, the present study supports feasibility and strong laboratory efficacy of DGC→MACS in a high-SDF population, but not comparative superiority over alternative algorithms.

That distinction is important because the sequencing question remains unresolved. Tavalaee et al. reported more favorable laboratory findings for MACS→DGC than DGC→MACS, especially with respect to active caspase-positive sperm and chromatin maturity [15]. Bucar et al. also found the greatest SDF reduction in a MACS→DGC→swim-up protocol, particularly in poorer-quality samples, whereas DGC→swim-up→MACS performed less well [14]. Berteli et al. likewise reported better recovery of high-quality spermatozoa when MACS preceded DGC [38]. Taken together, these data suggest that MACS-first approaches may be advantageous in selected laboratory settings. However, none of these studies provides definitive evidence that MACS-first processing yields better fertilization, pregnancy, or live-birth outcomes than DGC-first processing in routine clinical practice.

Conversely, some of the most clinically influential studies employed DGC before MACS. In the large retrospective cohort by Pacheco et al., adding MACS after DGC in couples with high SDF was associated with higher pregnancy and live-birth rates and lower miscarriage rates compared with DGC alone, while fertilization rates remained similar [9]. Sánchez-Martín et al. likewise reported lower miscarriage rates after MACS-based selection in men with high DNA fragmentation undergoing ICSI [10]. Mei et al. and Sahin Novin et al. reported favorable embryo-quality or transfer-efficiency outcomes in high-DFI populations treated with combined protocols [21,22]. These studies suggest that adding MACS to conventional preparation may have clinical value in selected patients, but they do not resolve whether DGC→MACS is better than MACS→DGC because their comparison groups were not sequence-matched [9,10,21,22].

A plausible biological explanation for the apparent advantage of this sequence, as suggested by the authors, is that performing DGC before MACS produces a more purified and homogeneous sperm fraction, thereby improving the efficiency of annexin V-mediated selection. By removing debris, round cells, and immotile spermatozoa before the magnetic step, the interaction between annexin V-conjugated beads and sperm cells with externalized phosphatidylserine may become more specific and effective. In this context, the cleaner background may promote stronger retention of apoptotic sperm and facilitate the recovery of a higher-quality non-apoptotic fraction from the column.

The available evidence therefore supports a more cautious interpretation than the current manuscript originally adopted. The literature supports the use of combined selection methods as promising tools to reduce SDF and potentially improve downstream reproductive outcomes in selected settings, but no large randomized head-to-head trial has directly compared DGC→MACS with MACS→DGC for fertilization, miscarriage, or live birth [9,11,15,23]. As emphasized in recent clinical guidance and updated evidence syntheses, the field remains limited by heterogeneous patient selection, inconsistent assay thresholds, and non-standardized laboratory workflows [9,11]. Consequently, the present study should not be framed as proving DGC→MACS superiority, but rather as adding prospective observational support for one effective sequence within an unresolved comparative field.

The difference between laboratory endpoints and reproductive endpoints deserves particular emphasis. SDF reduction is a relevant biological outcome, but it is not a surrogate that automatically predicts improved live birth. Embryo development, implantation, miscarriage, and delivery are influenced by multiple male and female factors, including oocyte repair capacity, female age, embryo selection practices, and the underlying cause of sperm DNA damage [1,11]. For this reason, strong laboratory findings can coexist with more modest or inconsistent clinical effects. This broader perspective is consistent with updated evidence indicating that elevated SDF has a clearer adverse association with IVF outcomes than with ICSI outcomes, and that evidence for live birth benefit after sperm-selection interventions remains less robust than many individual cohort reports imply [9,11].

The subgroup findings from both the literature and the current cohort may still have practical value if interpreted carefully. The present study enrolled only men with baseline SDF ≥ 30%, which makes high DNA fragmentation the clearest population in which the observed DGC→MACS benefit applies. Several reviewed studies also support the use of combined selection in men with high DFI, poor motility, and mixed sperm-parameter abnormalities [9,14,20,21,22,24,25]. Bucar et al. found greater SDF reduction in samples with lower vitality, membrane integrity, and progressive motility, while isolated teratozoospermia appeared to respond less favorably than asthenozoospermia or asthenoteratozoospermia [14]. Zhang et al. showed that DGC combined with MACS could identify viable sperm with low DNA fragmentation within an immotile sperm population [24]. Yang et al. extended potential utility to cryopreservation settings in men with poor sperm quality [26]. These findings are clinically useful for selective case identification, but they are derived from heterogeneous cohorts and should not be generalized as firm treatment rules.

The evidence on conventional semen parameters also points to a trade-off that should inform clinical decision-making. Some studies reported improvements in motility, viability, membrane integrity, and chromatin quality after combined preparation [19,21,24,25,37]. Others, particularly Çakar et al., raised concern about sperm loss and recovery after adding MACS, especially in oligozoospermic samples [16]. This suggests that the biological advantage of more stringent selection must be balanced against the practical risk of over-depletion in low-count ejaculates. In routine ART practice, the best preparation method may therefore depend not only on the degree of SDF but also on total sperm count, motility, diagnostic category, and the procedural demands of the planned treatment.

The mechanistic interpretation of sequencing effects should also remain measured. Several explanations have been proposed in the manuscript and in the cited literature, including the possibility that performing DGC first may alter membrane characteristics and affect subsequent annexin-binding patterns, or that MACS-first processing may remove apoptotic sperm before centrifugation-related stress can influence the sample [14,15,38]. These hypotheses are plausible and worth investigating, but they were not directly tested in the present study. Likewise, interpretations involving capacitation-related phosphatidylserine externalization, calcium-free buffer effects on motility assessment, or future fluorescence-guided selection platforms should be regarded as speculative methodological considerations rather than definitive explanations for current findings. Their value lies mainly in guiding future protocol refinement rather than in supporting present-day clinical claims.

The manuscript also highlights an additional point that deserves attention: combined sperm selection may influence not only SDF but also other markers of sperm quality, including chromatin compaction and chromosomal abnormality burden. Chi et al. reported reduced protamine deficiency after combined DGC and MACS compared with either method alone, and Delbes et al. showed enrichment of spermatozoa with improved chromatin quality after annexin V-based selection [19,20]. MACS has also been reported to reduce the proportion of spermatozoa carrying chromosomal abnormalities in selected samples, while DGC can decrease aneuploidy frequencies and the proportion of unbalanced sperm in specific patient groups [37,41,45,46]. These findings are biologically relevant, but their direct relationship to embryo competence and live birth remains incompletely defined and should not be overinterpreted.

The study has important limitations. First, the literature review was structured but not performed as a formal meta-analysis, and the included studies differed substantially in assay platform, baseline SDF thresholds, semen diagnoses, comparator groups, and processing protocols. Second, the original dataset was observational, single-sequence, and lacked a concurrent comparator arm, which prevents direct inference about superiority over DGC alone, MACS alone, or MACS→DGC. Third, the original cohort assessed laboratory improvement in SDF rather than reproductive endpoints such as embryo quality, pregnancy, miscarriage, or live birth. Fourth, female factors and embryo-related confounders were not integrated into the observational component. Reproductive endpoints (embryo quality, miscarriage, live birth) are mostly reported by retrospective or small prospective comparisons. Lastly, there exists no large randomized head-to-head DGC→MACS vs. MACS→DGC trial with live birth as primary outcome. These limitations are common in the broader SDF literature and help explain why the field still lacks universally accepted recommendations on optimal sperm-selection sequencing [9,11,22].

These constraints should temper the strength of clinical recommendations. This is explicitly acknowledged, but it limits the translational weight of the original dataset, especially given that SDF is not a validated surrogate for live birth in ICSI.

Despite these constraints, the present study contributes meaningfully to the current evidence base. It supports the view that combined sperm-selection approaches can substantially reduce SDF in selected infertile men, and it provides prospective blinded laboratory data showing that DGC→MACS is feasible and effective in a cohort with elevated baseline SDF. At the same time, the review underscores that the choice of sequence remains unsettled and that current recommendations should remain individualized rather than prescriptive. In clinical practice, combined DGC/MACS preparation may be considered particularly in men with persistently elevated SDF, especially when accompanied by asthenozoospermia or asthenoteratozoospermia, prior ART failure, or a clinical scenario in which additional selection is judged likely to outweigh sperm-loss risk [9,14,16,21,22,24].

Future studies should move beyond laboratory comparisons alone and directly address clinically meaningful endpoints. Well-designed randomized or carefully matched prospective comparative studies are needed to test DGC→MACS against MACS→DGC using standardized SDF assays, defined patient populations, and outcomes that include fertilization, embryo development, miscarriage, and live birth. Additional work is also needed to determine whether subgroup-directed selection strategies can identify men most likely to benefit from combined preparation and whether adjunctive markers of chromatin maturity or sperm aneuploidy can refine selection further. Until such evidence is available, combined DGC/MACS should be viewed as a promising but not yet definitively optimized strategy for the management of elevated SDF in ART.

### Strengths of This Study

The combination of a structured review and a prospective blinded observational cohort is a major strength. The cohort inclusion criterion (baseline SDF ≥ 30%) matches high-DFI thresholds used in several MACS trials and is consistent with guideline discussions of when SDF testing and sperm-selection interventions are most relevant [4,9,12,22].

## 7. Future Directions

Future studies should prioritize standardized, adequately powered head-to-head comparisons of DGC→MACS versus MACS→DGC, ideally incorporating both detailed laboratory endpoints and rigorous clinical outcomes such as live birth [11]. Additional work is also needed to better define which patient subgroups benefit most from combined selection and to quantify how improvements in SDF translate into embryo quality, miscarriage risk, and cumulative live-birth rates [4,6]. One interesting research step is focusing on the introduction of additional fluorescence-based microscope when performing MACS or alternatively a flow cytometry alongside MACS; this would require a multiparametric sperm-sorting methodology that combines apoptosis markers with chromatin/epigenetic markers, rather than relying only on annexin V. Technically, this implies either using a fluorescence microscope for “manual” selection or, more powerfully, a flow cytometer/sorter capable of detecting multiple fluorescent probes simultaneously and isolating the desired population in real time. For practical implementation, semen would first be prepared using density gradient centrifugation (DGC), with optional addition of MACS. An aliquot of the processed sample would then be stained using a rapid fluorescent chromatin assay (e.g., CMA3 or an SCSA-derived HDS marker) and examined under an epifluorescence microscope. The resulting chromatin pattern would guide selection, during ICSI, of spermatozoa that are morphologically normal and negative (or only weakly positive) for chromatin immaturity. Conceptually, this approach resembles MSOME, but it relies on fluorescence-based assessment rather than high magnification alone. When available, fluorescence-based flow cytometry equipped with sorting capability and multiple laser/filter configurations for various fluorochromes can rapidly quantify and discriminate populations differing in DNA fragmentation, chromatin maturity, aneuploidy, and apoptosis markers within the same sample, and can be coupled with cell sorting to physically recover the optimal subpopulation.

## 8. Conclusions

Combined DGC and MACS sperm preparation is a promising strategy for reducing sperm DNA fragmentation in selected infertile men. The reviewed literature and the present observational data support a substantial laboratory effect, particularly in men with elevated baseline SDF. However, the optimal sequencing strategy remains unresolved, and current evidence is insufficient to conclude that DGC followed by MACS is superior to MACS followed by DGC for major clinical ART outcomes.

However, the presented study protocol yielded excellent results when performing a DGC-MACS sequencing process in terms of reduction in SDF and improving semen parameters. This is consistent when DGC is considered as a coarse sieve that filters out debris and immature cells based on their weight and density, while MACS is like a precision magnet that specifically pulls out “bad apples” (apoptotic cells) that look healthy on the outside but are chemically flagged for disposal. Combining them ensures you have the heaviest, most mature, and chemically healthiest “fruit” for the final selection.

## Figures and Tables

**Table 1:** Included studies and their specifications.

	Study	Ref. Nr.	Study Type	Sample Size	Population	Primary Diagnosis
**1**	Tavalaee et al., 2012	[15]	Primary study	15 infertile men	Male infertility	Not specified
**2**	Degheidy et al., 2015	[25]	Primary study	36 patients	Varicocele patients	Varicocele
**3**	Sanchez-Martin et al., 2017	[10]	Retrospective clinical cohort study	305 couples	High SDF (≥30%)	High DNA fragmentation
**4**	Toishibekov et al., 2021	[33]	Primary study	63 patients	Primary infertility	Oligoasthenoteratozoospermia
**5**	Salehi Novin et al., 2023	[21]	Primary study	30 couples	Male-factor infertility	High DFI (>30%)
**6**	Zhang et al., 2018	[24]	Primary study	16 patients	Male-factor infertility	Asthenozoospermia
**7**	Bucar et al., 2015	[14]	Experimental comparative study	100 semen samples	Various diagnoses	Teratozoospermia, asthenozoospermia
**8**	Chi et al., 2016	[19]	Experimental comparative study	458 semen samples	Various diagnoses	Asthenozoospermia, teratozoospermia
**9**	Yang, S et al., 2023	[26]	Primary study	16 patients	Poor sperm quality	Asthenozoospermia, teratozoospermia
**10**	Pacheco et al., 2020	[9]	Retrospective clinical cohort	724 cycles	High SDF (>20%)	High DNA fragmentation
**11**	Lee et al., 2010	[17]	Primary study	60 couples	Unexplained infertility	Unexplained infertility with IUI failures
**12**	Delbes et al., 2013	[20]	Primary study Experimental study of sperm samples	42 patients	Various diagnoses	Normozoospermic, asthenoteratozoospermic, teratozoospermia
**13**	Fang et al., 2018	[34]	Primary study	Not specified	IVF patients	Various
**14**	Mei et al., 2022	[22]	Primary study	86 patients	High DFI (≥30%)	High DNA fragmentation
**15**	Notrica et al., 2013	[35]	Primary study	74 couples	Male-factor infertility	Teratozoospermia with high DNA fragmentation
**16**	Tezcan et al., 2020	[36]	Primary study	17 couples	Unexplained infertility	Unexplained infertility
**17**	Esbert et al., 2017	[37]	Prospective study	16 males	Abnormal FISH	Chromosomal abnormalities
**18**	Berteli et al., 2017	[38]	Prospective comparative sequence study	15 semen samples	Infertility assessment	Fertile men
**19**	Said et al., 2006	[18]	In vitro model MACS	35 samples	Healthy donors	Hamster oocyte penetration assay/hamster oocyte ICSI, SCD in spermatozoa
**20**	Ziarati et al., 2019	[23]	Prospective RCT	62 semen samples	ICSI candidates	Male infertility
**21**	Çakar et al., 2016	[16]	Primary study	20 donors	Normozoospermic, oligozoospermic	Oligozoospermia
**22**	Bibi et al., 2023	[39]	Prospective study	385 couples	Isolated teratozoospermia	Teratozoospermia (allocation to 4 sperm prep methods)
**23**	Merino-Ruiz et al., 2019	[40]	Experimental study	92 couples	IVF/ICSI patients	Various factors
**24**	El Fekih et al., 2022	[41]	Experimental study	6 men (cryopreserved sperm samples)	infertile male	Not mentioned, high sperm DNA fragmentation
**25**	Falquet Guillem M et al., 2026	[11]	Systematic review and meta-analysis	41 studies	comparing MACS vs. conventional sperm selection in MAR	Male infertility/high sperm DNA fragmentation
**26**	Dirican E et al., 2008	[42]	Prospective comparative ICSI study	122 MACS before ICSI vs. 74 DCG before ICSI	ICSI patients	Male infertility by OAT, no data for sperm defragmentations

**Table 2:** Overview of treatment protocols: DGC→MACS, MACS→DGC, swim-up combinations, and comparative protocols.

Protocol Type	Studies	Key Details
DGC→MACS (DGC first)	Degheidy et al. [25]; Sánchez-Martín et al. [10]; Toishibekov et al. [33]; Zhang et al. [24]; Chi et al. [19]; Pacheco et al. [9]; Lee et al. [17]; Delbes et al. [20]; Esbert et al. [37]; Said et al. [18]; Bibi et al. [39]; Merino-Ruiz et al. [40]; Berteli et al. [38]	DGC followed by MACS selection
MACS→DGC (MACS first)	Tavalacee et al. [15]; Salehi Novin et al. [21]; Fang et al. [34]; Ziarati et al. [23]; Tezcan et al. [36]; Berteli et al. [38]; Dirican et al. [42]	MACS followed by DGC
Combined with swim-up	Bucar et al. [14]; Mei et al. [22]	DGC-MACS-SU or MACS-DGC-SU sequences
Comparative protocols	Berteli et al. [38]; Çakar et al. [16]	Multiple protocol comparisons

Treatment protocols evaluated in the reviewed studies. DGC→MACS (DGC first) includes protocols in which density gradient centrifugation was followed by MACS; MACS→DGC (MACS first) includes protocols in which MACS preceded DGC; combined-with-swim-up protocols include DGC→MACS→SU or MACS→DGC→SU sequences; and comparative protocols refer to studies directly comparing multiple preparation strategies.

**Table 3:** Effects on DFI after different sequence protocols for DGC, MACS, SU and combined treatment protocols.

Study (Arm)	Ref. Nr.	DFI Assay	Pre-Treatment DFI (%)	Post-Treatment DFI (%)	Absolute Reduction (pp)	% Reduction	Statistical Significance	Notes
Tavalaee et al., 2012;→DGC	[15]	TUNEL	29.72 ± 3.41	21.27 ± 3.47.	8.45	28.4%	*p* < 0.05 vs. control	N = 15 semen samples, grouped in OAT (n = 3), OA (n = 2), asthenozoospermic (n = 3) and normozoospermic (n = 7)
Tavalaee et al., 2012; →MACS	[15]	TUNEL	29.72 ± 3.41	21.72 ± 3.41	8.00	26.9%	*p* < 0.05 vs. control	
Tavalaee et al., 2012;DGC→MACS	[15]	TUNEL	29.72 ± 3.41	17.63 ± 3.72	12.09	40.7%	*p* < 0.05 vs. control	
Tavalaee et al., 2012; MACS→DGC	[15]	TUNEL	29.72 ± 3.41	15.27 ± 3.49	14.45	48.6%	*p* < 0.05 vs. control	
Degheidy et al., 2014; DGC→MACS	[25]	TUNEL	12.43	9.61	2.82	22.7%	*p* < 0.05	Pre = post-DGC aliquot before MACS; post = after MACS.
Toishibekov et al., 2021; Annexin− fraction	[33]	SCSA (DFI)	32.40	10.50	21.90	67.6%	*p* < 0.01 (vs. original)	Pre = raw semen; post = annexin V− after processing (double DGC + MACS).
Chi et al., 2016; →DGC	[19]	SCD (Halosperm)	11.50	8.10	3.40	29.6%	*p* < 0.05 vs. control	Pre = raw semen control.
Chi et al., 2016;→MACS (Annexin−)	[19]	SCD (Halosperm)	11.50	7.40	4.10	35.7%	*p* < 0.05 vs. control	Pre = raw semen control.
Chi et al., 2016: →DGC+MACS	[19]	SCD (Halosperm)	11.50	4.10	7.40	64.3%	*p* < 0.05 vs. control; lower than DGC or MACS alone	Pre = raw semen control.
Zhang et al., 2017; →DGC	[24]	TUNEL	9.56	5.25	4.31	45.1%	*p* < 0.05 vs. control	Pre = unprocessed control.
Zhang et al., 2017; →DGC+MACS	[24]	TUNEL	9.56	2.75	6.81	71.2%	*p* < 0.05 vs. control and vs. DGC; overall *p* < 0.01	Pre = unprocessed control.
Tezcan et al., 2020; →DG→MACS+DG	[36,38]	TUNEL	80.12	41.00	39.12	48.8%	*p* < 0.01	Both values are post-processing (DG vs. MACS+DG); raw baseline not reported.
Berteli et al., 2017: →DGC	[38]	TUNEL	24.00	10.00	14.00	58.3%	processed groups differ (*p* < 0.05)	Values are medians; baseline not part of processed-group comparison.
Berteli et al., 2017:→DGC→MACS	[38]	TUNEL	24.00	6.00	18.00	75.0%	processed groups differ (*p* < 0.05)	Values are medians; baseline not part of processed-group comparison.
Berteli et al., 2017; →MACS→DGC	[38]	TUNEL	24.00	4.00	20.00	83.3%	processed groups differ (*p* < 0.05)	Values are medians; baseline not part of processed-group comparison.
Berteli et al., 2017; →MACS	[38]	TUNEL	24.00	8.00	16.00	66.7%	processed groups differ (*p* < 0.05)	Values are medians; baseline not part of processed-group comparison.
Bucar et al., 2015; →DGC→SU	[14]	TUNEL	4.30	1.10	3.20	74.4%	T0 vs. T1: *p* < 0.05	T0 = raw semen; T1 = after DGC+SU; n = 20;
Bucar et al., 2015; →DGC→MACS→SU	[14]	TUNEL	5.00	1.00	4.00	80.0%	T0 vs. T1: *p* < 0.05	T0 = raw semen; T1 = after DGC+MACS+SU; n = 20;
Bucar et al., 2015; →DGC→SU→MACS	[14]	TUNEL	8.20	4.20	4.00	48.8%	T0 vs. T1: *p* < 0.05; less efficient vs. other groups: *p* < 0.05	T0 = raw semen; T1 = after DGC+SU+MACS; n = 20;
Bucar et al., 2015; →MACS→DGC→SU	[14]	TUNEL	5.50	1.10	4.40	80.0%	T0 vs. T1: *p* < 0.05	T0 = raw semen; T1 = after MACS+DGC+SU; n = 20;
Bucar et al., 2015; →MACS→SU	[14]	TUNEL	4.30	1.20	3.10	72.1%	T0 vs. T1: *p* < 0.05	T0 = raw semen; T1 = after MACS+SU; n = 20;
Bibi et al., 2023; →DGC	[39]	SCD	20.90	14.70	6.20	29.7%	Post-prep comparison *p* = 0.01;	TZS Baseline SDF comparable across groups (*p* = 0.68) DGC-MACS lower than DGC & SU
Bibi et al., 2023; →SU	[39]	SCD	23.10	14.50	8.60	37.2%	Post-prep comparison *p* = 0.01;	DGC-MACS lower than DGC & SU
Bibi et al., 2023; →DGC→SU		SCD	25.15	14.20	10.95	43.5%	Post-prep comparison *p* = 0.01;	DGC-MACS lower than DGC & SU
Bibi et al., 2023; →DGC→MACS	[39]	SCD	25.30	12.30	13.00	51.4%	(ANOVA *p* = 0.01)	Lower than DGC & SU after preparation

Sperm DNA fragmentation outcomes after different sperm-preparation protocols, including density gradient centrifugation (DGC), magnetic-activated cell sorting (MACS), swim-up (SU), and combined sequencing strategies. The table summarizes the assay used, pre- and post-treatment DFI values, absolute reduction, percentage reduction, statistical significance, and study-specific notes for each treatment arm. Teratozoospermia (TZS); sperm chromatin dispersion (SCD).

**Table 4:** Summary of study-level effects of combined sperm-preparation protocols on sperm parameters in different clinical conditions.

Study	Parameter		Pre-Treatment	Post-Treatment	Change
Toishibekov et al., 2021; [33]	Motility		32.7 ± 5.9%	47.2 ± 6.3%	+14.5%
Zhang et al., 2018; [24]	Live spermatozoa		65.88 ±12.77%	85.81 ± 5.2%	+19.93%
	Membrane integrity		52.5 ± 12.21%	81.81 ± 5.29%	+29.31%
Esbert et al., 2017; [37]	Progressive motility		Baseline	Sign. increased	*p* < 0.001
Salehi Novin et al., 2023; [21]	Progressive motility		Not reported	Sign. improved	Sign. increase
	Normal morphology		Not reported	Sign. improved	Sign. increase
Cakar Zeynep et al., 2016; [16]	Sperm concentration in NZS	SU	43.0 ± 21.3	21.1 ± 11.9	−50.9%
		SU+MACS		8.3 ± 6.1	−80.7%
		DGC		20.0 ± 11.2	−53.5%
		DGC+MACS		12.1 ± 7.6	−71.9%
	Sperm concentration in OZS	SU	9.6 ± 3.8	3.8 ± 2.9	−60.4%
		SU+MACS		2.5 ± 2.1	−74.0%
		DGC		6.2 ± 2.8	−35.4%
		DGC+MACS		3.5 ± 3.4	−63.5%
Cakar Zeynep et al., 2016; [16]	Rap. prog. motility in NZS	SU	19.6 ± 14.3	9.3 ± 6.9	−52.6%
		SU+MACS		4.1 ± 2.7	−79.1%
		DGC		6.0 ± 4.5	−69.4%
		DGC+MACS		2.5 ± 1.8	−87.2%
	Rap. prog. motility in OZS	SU	2.4 ± 0.8	1.4 ± 1.0	−41.7%
		SU+MACS		0.02 ± 0.04	−99.2%
		DGC		0.9 ± 0.8	−62.5%
		DG+MACS		1.5 ± 2.5	−37.5%

Legend: Normozoospermia (NZS); Oligozoospermia (OZS), density gradient centrifugation sperm selection (DGC); magnetic-activated cell sorting (MACS); swim-up (SU).

**Table 5:** Embryological and clinical ART outcomes after DGC, DGC-MACS, and related sperm-selection protocols, including fertilization, embryo quality, pregnancy, miscarriage, and live-birth rates.

Study	Population/Setting	Outcome Measure	DGC Only	DGC-MACS	Statistical Significance
Salehi Novin et al., 2023; [21]	Male-factor infertile couples with high DNA fragmentation; DGC vs. DGC-MACS	Fertilization rate	73.11%	72.07%	N.s.
		Top-quality embryos (d3)	51.47%	72.5%	*p* < 0.05
		Blastocyst rate	48%	69.69%	Significant
Pacheco et al., 2020; [9]	High SDF ICSI cycles; MACS-based selection	Fertilization rate	73.3%	75.1%	*p* = 0.13
		Pregnancy rate	51.5%	60.7%	*p* = 0.014
		Miscarriage rate	20.6%	14.7%	*p* = 0.034
		Live-birth rate	31.2%	47.4%	*p* = 0.001
Ziarati et al., 2019; [23]	Infertile couples, male-factor ICSI	Fertilization rate	Control	No significant difference	n.s.
		High-quality embryos	Control	Significantly higher	Significant
		Pregnancy rate	Control	Significantly higher	Significant
Mei et al., 2021; [22]	High-DFI IVF/ICSI patients	Live-birth rate (first cycle)	53.9%	63.2%	Trend toward improvement
		Cumulative live-birth rate	70.7%	79.5%	Trend toward improvement
		Transfer cycles per retrieval	1.6 ± 0.8	1.2 ± 0.5	Significant reduction
Notrica et al., 2013; [35]	Teratozoospermic sperm with highly fragmented DNA, DGC + annexin V–MACS	Fertilization rate (ICSI)	84.2 ± 4.4%	77.4 ± 3.0%	*p* = 0.4
		Pregnancy rate (ICSI)	42.9%	39.0%	*p* = 1
Bibi et al., 2023; [39]	Comparative ART outcomes across semen preparation methods in 385 infertile couples by isolated teratozoospermia	Pregnancy rate	DGC-SU 48%	52.5%	*p* < 0.01
		Cleavage rate	DGC 81% for SDF < 20% 85% for SDF > 20%	Significant increase in DGC-MACS group: 93% for SDF < 20% 87% for SDF > 20%	The paper reports a significant improvement in cleavage rate for DGC-MACS.
		Implantation rate	Comparable across groups 21% for SDF < 20% 38% for SDF > 20%	Comparable across groups 36% for SDF < 20% 35% for SDF > 20%	n.s. difference in implantation rate was observed.
		Fertilization rate (2PN)	66%	70%	73% for SU 69% for DGC-SU
		Fertilization rate (2PN) by SDF > 20%	73%	58%	n.s.

**Table 6:** Baseline factors associated with with the response to sperm-selection protocols.

Factor	Findings	Study
Initial sperm quality	Lower progressive motility, vitality, and membrane integrity were associated with greater SDF reduction after MACS-based selection.	Bucar et al., 2015; [14]
Semen diagnosis	Teratozoospermic samples showed lower SDF reduction rates than asthenozoospermic or asthenoteratozoospermic samples.	Bucar et al., 2015 [14]
Baseline DFI	Men with DFI > 30% showed reduced DFI and improved embryology outcomes after MACS-DGC.	Salehi Novin et al., 2023 [21] Toishibekov et al., 2021
PLCζ expression	Higher PLCz1 expression was observed in MACS-DGC-selected sperm and coincided with better early embryo development. Infertile men generally have lower PLCζ expression than fertile controls.	Salehi Novin et al., 2023 [21] Parrella et al., 2024 [47]
Apoptotic markers	Sperm apoptotic markers correlated with sperm quality and ART outcomes, but were not fully validated as stand-alone predictors of response.	Said et al., 2006 [18]
Morphology baseline	DGC-MACS can recover viable sperm with lower DNA fragmentation from immotile samples.	Zhang et al., 2018 [24]

Summary of study-level evidence examining associations between baseline semen quality, diagnostic category, DNA fragmentation index, phospholipase C zeta expression, apoptotic markers, and sperm phenotype and the magnitude of SDF reduction or subsequent embryological outcomes following DGC, MACS, DGC–MACS, and related sperm-selection protocols. These associations are exploratory and should not be interpreted as validated independent predictors of treatment response. **ART**, assisted reproductive technology; **DFI**, DNA fragmentation index; **DGC**, density gradient centrifugation; **MACS**, magnetic-activated cell sorting; **PLCζ**, phospholipase C zeta; **SDF**, sperm DNA fragmentation.

**Table 7:** Sperm DNA fragmentation (SDF) before and after DGC-MACS treatment (n = 67).

Parameter	Mean ± SD	Range	Median
Native sperm SDF	41.61 ± 11.2%	30–78%	38%
Absolute SDF reduction	19.42 ± 9.4%	4–43%	19%
Statistical significance	*p* < 0.0001		

Values are presented as mean ± SD unless otherwise indicated.

## Data Availability

The original contributions presented in this study are included in the article. Further inquiries can be directed to the corresponding author.
